# Identification of a novel mild isolate of areca palm necrotic spindle-spot virus (ANSSVm) lacking two cysteine proteases (HC-Pro1 and HC-Pro2)

**DOI:** 10.3389/fmicb.2025.1553892

**Published:** 2025-07-15

**Authors:** Hang Tan, Xianmei Cao, Hongxing Wang, Ruibai Zhao, Xi Huang

**Affiliations:** ^1^School of Breeding and Multiplication (Sanya Institute of Breeding and Multiplication), Hainan University, Sanya, China; ^2^School of Life and Health Sciences, Hainan University, Haikou, China

**Keywords:** *Potyviridae*, *Arepavirus*, areca palm necrotic spindle-spot virus (ANSSV), mild isolate, infectious clone, viral movement

## Abstract

Areca palm necrotic spindle-spot virus (ANSSV) encodes two tandem cysteine proteases, HC-Pro1 and HC-Pro2, at its N-terminal region, essential for maintaining viral infectivity. Deletion of either protease from an infectious clone abolishes infectivity. We report a mild ANSSV isolate, designated ANSSVm, from areca palm leaves exhibiting mild chlorosis. ANSSVm has a truncated genome of 7,868 nt, lacking both HC-Pro1 and HC-Pro2. An infectious ANSSVm clone established systemic infection in *Nicotiana benthamiana* through agroinfiltration but caused no symptoms in inoculated plants. Nucleotide polymorphisms in ANSSVm clones influenced infectivity, and residue variations of coat protein (CP) and P3 were identified as critical for intercellular movement. However, amino acid substitutions in ANSSVm CP did not alter its interactions with host proteins associated with viral movement. Further research is needed to uncover host factors and interactions essential for the systemic spread of potyvirids, presenting opportunities for targeted viral control strategies in plants.

## Introduction

Areca palm (*Areca catechu* L.) is a crucial crop in Southeast Asia due to its significant economic value. The growing demand for areca nuts has driven rapid expansion of areca palm plantations, with Hainan Province in China now cultivating over 2,00,000 hectares. However, large-scale monoculture reduces biodiversity, thereby increasing the risk of infectious diseases (Johnson et al., 2015; Keesing et al., 2010). Areca palm is vulnerable to various pathogens, including bacteria, fungi (To-Anun et al., 2009; Wang et al., 2019), phytoplasmas (Ramaswamy et al., 2013; Kanatiwela-De Silva et al., 2015), and viruses (Zhang et al., 2022; Wang et al., 2020; Khan et al., 2023; Cao et al., 2021; Cao et al., 2024). Among these, viral diseases pose the most severe threat to areca palm cultivation.

Areca palm necrotic spindle-spot virus (ANSSV) was the first potyvirid identified in areca palm (Yang et al., 2018). Its 9,437 nt positive-sense single-stranded RNA genome encodes a 3,019 aa polyprotein. Another potyvirid, areca palm necrotic ringspot virus (ANRSV), associated with areca palm necrotic ringspot disease (ANRSD), was subsequently identified (Yang et al., 2019). Phylogenetic analysis and sequence comparison placed ANSSV and ANRSV within the newly proposed genus *Arepavirus* of the family *Potyviridae* (Yang et al., 2019). Infectious clones of ANRSV and ANSSV were shown to rapidly multiply and induce severe symptoms in *N. benthamiana*. Functional hybrid viruses constructed by swapping genomic elements between the two species revealed that substituting regions of 5’-UTR-HCPro1-HCPro2 or CI effectively supported replication and systemic infection of ANRSV, while substitutions of P3-7K, 9K-NIa, or NIb-CP-3’-UTR abolished infectivity. Furthermore, ANRSV effectively excluded ANSSV in co-infection and super-infection assays, confirming their close genetic relationship (Wang et al., 2021).

ANSSV encodes two tandem cysteine proteases, HC-Pro1 and HC-Pro2, at its N-terminal region. These two cysteine proteases have distinct functional roles and HC-Pro2 acts as a viral suppressor of RNA silencing (VSR). Deletion of HC-Pro1 or HC-Pro2 in infectious clones abolishes systemic infection, and neither deletion can be rescued by counterparts from turnip mosaic virus (TuMV) (Qin et al., 2020). Similarly, HC-Pro2 of ANRSV is essential for infection, forming complexes with viral factors such as CI and CP, as well as the host Rubisco small subunit (RbCS), to facilitate plasmodesma-localized intercellular movement (Qin et al., 2024). These findings emphasize the critical roles of HC-Pro1 and HC-Pro2 in viral infection.

Yellow leaf disease (YLD) represents a major challenge to areca palm cultivation, causing significant yield losses and economic damage (Khan et al., 2023; Wang et al., 2020). The absence of resistant cultivars highlights the urgent need for alternative strategies to combat viral diseases. Traditional Agrobacterium-mediated transformation methods face limitations in areca palm, a perennial tropical tree (Wang et al., 2023; Nguyen et al., 2015). Virus-induced gene silencing (VIGS) offers a potential solution for gene silencing in plants with transformation difficulties (Baulcombe, 2015; Burch-Smith et al., 2004; Wu et al., 2022; Liu et al., 2024). In our efforts to develop a VIGS system for areca palm, we recently identified a mild isolate of ANSSV, termed ANSSVm, from areca palm leaves with mild chlorosis symptoms. ANSSVm has a truncated genome of 7,868 nt, lacking HC-Pro1 and HC-Pro2. The infectious clone of ANSSVm successfully caused systemic infection in *N. benthamiana* through agroinfiltration. This discovery provides a unique opportunity to explore novel mechanisms of viral movement and systemic infection of potyvirids. Additionally, ANSSVm did not induce visible symptoms on host plant, offering a promising foundation for establishing a VIGS system in areca palm, potentially accelerating the development of virus-resistant cultivars.

## Materials and methods

### Virus source and plant materials

An areca palm leaflet exhibiting mild chlorotic spots from the palm in which ANSSV-HNBT was initially discovered (Yang et al., 2018) was collected in 2021 and used for construction of ANSSVm clones. Another areca palm seedling exhibiting severe necrotic spindle-spots indicative of ANSSV infection was found in Nanlin Town, Baoting County, Hainan, China and used for construction of a full-length ANSSV clone (ANSSV1). Wild-type and transgenic 16c *N. benthamiana* seedlings were germinated and maintained in a greenhouse under controlled conditions of 22 ± 2°C with a 16 h light/8 h dark photoperiod.

### Sequencing and annotation of the viral genome

To determine the complete viral genome sequence, overlapping primer sets covering nearly the entire genome (Supplementary Table S1) were designed based on the reference genome of ANSSV-HNBT (GenBank NC_040836). Rapid Amplification of cDNA Ends (RACE) was performed to validate the 5’- and 3’-terminal sequences using the 5’/3’ RACE Kit, 2nd Generation (Roche, Indianapolis, IN), following the manufacturer’s protocol. PCR amplification was conducted with PrimeSTAR^®^ GXL DNA Polymerase (TaKaRa, Dalian), and the products were incubated with Taq polymerase at 72°C for 10 min before ligation into the pMD-19T vector (TaKaRa, Dalian). At least three independent positive clones for each fragment were subjected to Sanger sequencing (Sangon Biotech, China). The resulting overlapping sequences were analyzed and assembled into a consensus full-length genome using SeqMan Pro 7.1.0 (Lasergene, GATC Biotech). Proteolytic cleavage sites and mature proteins encoded by the polyprotein were annotated as described previously (Yang et al., 2018). Sequence identities for amino acids (aa) and nucleotides (nt) were calculated using DNAMAN software with default parameters (Lynnon BioSoft).

### Construction of infectious cDNA clones and site-directed mutagenesis

GFP-tagged infectious cDNA clones of ANSSVm were constructed following the approach used for the ANSSV-HNBT GFP expression vector (Qin et al., 2020), with an additional Hepatitis delta virus (HDV) self-cleaving ribozyme (Been, 1994) placed upstream of the nopaline synthase (NOS) terminator to ensure precise cleavage at the poly(A) tail. The initial binary vector p35SRz, derived from pCAMBIA3300, contained a duplicated CaMV 35S promoter, a multiple cloning site flanked by *Stu*I and *Sma*I, the HDV ribozyme, and a NOS terminator. This design facilitated the generation of viral transcripts with accurate 5’ and 3’ termini after the full-length viral cDNA was subcloned between *Stu*I and *Sma*I (Scholthof, 1999). To insert an intron into the P3-coding region, overlap PCR (Horton et al., 1989) was employed using intron 2 of the *Phaseolus vulgaris* nitrite reductase gene (*PvNIR*, GenBank U10419). This step generated a chimeric PCR product of 3.9 kb covering the 5’-terminal region of the viral genome. The chimeric product, two additional viral fragments (3rd and 4th), the GFP coding sequence, and the vector backbone (digested with *Stu*I and *Sma*I) were assembled into a full-length clone using a recombination-based one-step assembly method (Vazyme, China). The primers used in this work were listed in Supplementary Table S1. The *Escherichia coli* strain Stbl2 was used to stabilize the construct. Randomly selected clones were screened preliminarily by *Hin*dIII digestion and further verified by next-generation sequencing (Sangon Biotech, China). Site-directed mutagenesis was performed using a recombination-based mutagenesis kit (Vazyme, China) with primers listed in Supplementary Table S1. All point mutant constructs were confirmed by Sanger sequencing prior to use.

### Agroinfiltration

*N. benthamiana* seedlings at the four/five true-leaf stage were used for agroinfiltration. Procedures were as previously described (Chen et al., 2012) with minor modifications. Briefly, a single colony of *Agrobacterium tumefaciens* GV3101 transformed with the desired construct was inoculated into 10 mL LB media supplemented with 50 μg/mL of kanamycin, 25 μg/mL of rifampicin, 20 μM acetosyringone (AS), and 10 mM N-morpholinoethanesulfonic acid (MES), and grown overnight at 28°C with shaking at 200 rpm until an OD_600_ of 0.6–1.0 was reached. *A. tumefaciens* was pelleted by centrifugation at 3,000 × *g* for 5 min and resuspended in 10 mL suspension solution (10 mM MgCl_2_ and 10 mM MES). The resuspended bacteria were then centrifuged at 3,000 × *g* for 5 min and the pellet was resuspended in infiltration buffer (10 mM MgCl_2_, 200 μM AS, and 10 mM MES, pH 5.6) to a final OD_600_ of 1.0. This suspension was kept in dark for 2–3 h before being infiltrated into fully expanded leaves using needleless 1 mL syringes. The infectious clones of ANSSVm were co-infiltrated in equal proportions with a plasmid expressing the p19 suppressor of tomato bushy stunt virus (p35S-p19^*TBSV*^) to enhance the inoculation efficiency.

### RNA extraction and reverse transcription-quantitative PCR

Total RNA was extracted from *N. benthamiana* and areca palm leaves using the Tiangen RNAprep Pure Plant Plus Kit (Tiangen Biotech, China) according to the manufacturer’s instructions. Reverse transcription of the total RNA was conducted using the Thermo Scientific RevertAid First Strand cDNA Synthesis Kit (Thermo Fisher Scientific, United States) following the manufacturer’s instructions. Quantitative PCR (qPCR) was performed using the TransStart Tip Green qPCR SuperMix (TransGen Biotech, China). The *NbActin* gene was used as the internal control. At least three biological replicates were used for all experiments and the relative gene expression levels were calculated using the 2^–ΔΔ^*^Ct^* method (Livak and Schmittgen, 2001). Primers used for RT-qPCR were listed in Supplementary Table S1.

### Protein extraction and western blotting

Total protein was extracted from liquid nitrogen-lyophilized leaf tissue using extraction buffer [9 M urea, 50 mM Tris-HCl (pH 6.8), and 4.5% sodium dodecyl sulfate (SDS), supplemented with 7.5% 2-Mercaptoethanol before use]. The supernatant was collected by centrifugation at 12,000 × *g* for 10 min at 4°C. For Western blotting assays, protein samples were separated by 12% SDS-PAGE, electroblotted onto a polyvinylidene difluoride (PVDF) membrane, and detected by immunoblotting. The blots were treated with SuperSignal West Pico PLUS (Thermo Fisher Scientific, United States) and visualized using the BG-gdsAUTO710 Mini Imaging System (Baygene, China).

### Bimolecular fluorescence complementation and confocal microscopy

BiFC plasmids were constructed by using recombination-based one-step assembly (Vazyme, China). The coding sequence (CDS) of interest with compatible cloning adaptors (15–25 bp overlap) were amplified with specific primers (Supplementary Table S1) and cloned to the linearized empty vectors pFGC-YC155 (35S: C-terminal YFP, cYFP) or pFGC-YN173 (35S: N-terminal YFP, nYFP) to generate the desired constructs. All constructs were verified by Sanger sequencing. Three *A. tumefaciens* cultures transfected with cYFP-derived construct, nYFP-derived construct, and p35S-p19^*TBSV*^ expressing the p19 silencing suppressor, respectively, were mixed in a ratio of 1:1:0.3 prior to infiltration. The epidermal cells of infiltrated leaves were examined for YFP fluorescence using the confocal laser scanning microscope LSM 780 (Carl Zeiss) with scan settings for YFP of Ex at 514 nm and Em at 565–585 nm. Images were processed with the LSM software (Carl Zeiss). Statistical analysis of the viral intercellular movements was analyzed by the ImageJ software.

## Results

### Sequencing and genome analysis of a novel ANSSV isolate

Attempts to clone the complete coding sequence of HC-Pro2 from an areca palm leaf sample exhibiting mild chlorotic spots typical of ANSSV infection yielded no amplicons. However, successful amplification and sequencing of the full-length CP from the same cDNA preparation confirmed the presence of ANSSV in the sample. To identify this novel ANSSV isolate, RT-PCR and RACE experiments were conducted to sequence the entire genome. Using the ANSSV reference genome (ANSSV-HNBT, GenBank NC_040836), six overlapping back-to-back primer sets were designed to cover the entire genome (Supplementary Table S1). While the expected bands for four 3’-proximal primer sets spanning CI to CP were successfully amplified, the first 5’-proximal primer set, covering the complete HC-Pro1 and HC-Pro2 in tandem, produced an unexpected band of approximately 0.5 kb—much shorter than the expected 2 kb. Sequencing of this 0.5 kb product revealed that the 3’ portion of HC-Pro1, the complete HC-Pro2, and a few amino acids at the N-terminus of P3 were missing, resulting in a 1.5 kb gap compared to the reference genome. Consistently, the second 5’-proximal primer set, with its forward primer located within the C-terminal region of HC-Pro2, failed to produce any visible bands. Additional primers targeting regions inside and outside the gap were designed and tested in various combinations to rule out PCR artifacts (data not shown). Sequencing results from all amplicons confirmed that the 1.5 kb gap was consistently present, with a stable joint site linking the truncated HC-Pro1 and P3. To validate the genomic ends, 5’ RACE and 3’ RACE experiments were performed. All obtained sequences were assembled into the consensus genome of this mild ANSSV isolate, designated as ANSSVm (GenBank accession PQ776890).

**FIGURE 1:**
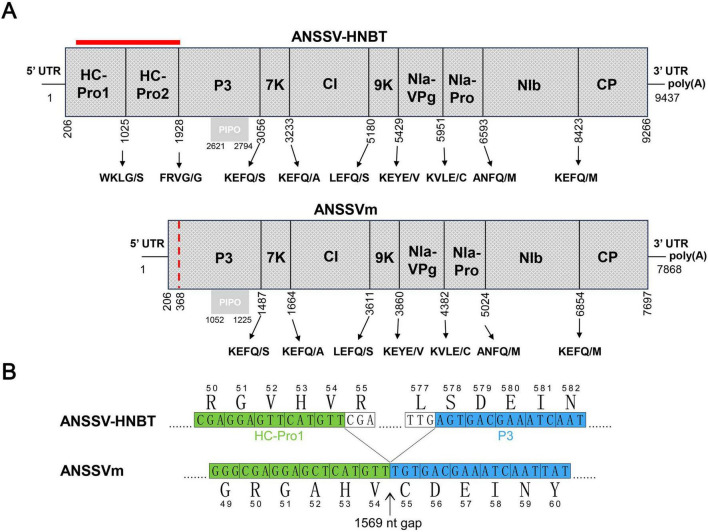
The genome organization of ANSSVm. **(A)** Comparison of genome organizations of ANSSV-HNBT and ANSSVm. The genome organization of ANSSVm is almost identical with that of ANSSV-HNBT (NC_040836) except for a 1,569 nt gap encompassing the 3’ majority of HC-Pro1, the complete HC-Pro2, and a few amino acids at the N-terminus of P3 (indicated by red bar). The red dotted line indicates the junction between the residual HC-Pro1 and P3 in ANSSVm. ANSSV encodes a large polyprotein that is proteolytically processed into 10 mature proteins by HC-Pro1, HC-Pro2, or NIa-Pro proteinases. The polyprotein encoded by ANSSVm is suggested to produce eight mature proteins via proteolysis by NIa-Pro. The gray box represents PIPO that is derived from RNA polymerase slippage in the P3-coding region. **(B)** Demarcation of the junction between the residual HC-Pro1 and P3 in ANSSVm. The partial HC-Pro1 of ANSSVm is suggested to be 54 aa in length, as its C-terminal end ^51^GAHV^54^ matched ^51^GVHV^54^ of the full-length HC-Pro1. The partial P3 of ANSSVm is presumed to lack three amino acids at the N-terminus, as its N-terminal end ^55^CDEIN^59^ matched ^578^SDEIN^582^ of the full-length P3. Nucleotide sequences were presented in form of triple codes along with the translated aa sequences. Numbers indicate the relative positions of amino acids in the complete genome of ANSSV-HNBT or ANSSVm. Aligned sequences encoding HC-Pro1 and P3 were colored green and blue, respectively.

The complete genome of ANSSVm is 7,868 nucleotides (nt) long [excluding the poly(A) tail] and encodes a polyprotein of 2,496 amino acids (aa). Its genomic organization is nearly identical to that of ANSSV-HNBT, with identical 5’ UTR and 3’ UTR lengths and coding sequences for the proteolytically cleaved mature proteins, except for a 1,569 nt deletion encompassing the 3’ portion of HC-Pro1, the entire HC-Pro2, and a few amino acids of the P3 protein (Figure 1A). Pairwise alignment of ANSSVm with ANSSV-HNBT revealed 99.58% nt identity (33 mismatches) between their genomes and 99.40% aa identity (15 mismatches) between their polyproteins. The joint site between the truncated HC-Pro1 and P3 in ANSSVm was analyzed using nt and aa alignments (Figure 1B). The truncated HC-Pro1 was determined to be 54 aa long, with its C-terminal sequence (^51^GAHV^54^) matching ^51^GVHV^54^ of the full-length HC-Pro1. The partial P3 lacked three amino acids at its N-terminus, with its sequence starting from ^55^CDEIN^59^ aligning with ^578^SDEIN^582^ of the full-length P3 in ANSSV-HNBT (Figure 1B).

The HC-Pro of potyvirids is known to function as a self-processing protease, with its C-terminal domain mediating proteolytic cleavage. In ANSSV HC-Pro1, the conserved cysteine protease domain is located upstream of the cleavage site ^273^G/V^274^ (Qin et al., 2020). This domain is absent in the residual HC-Pro1 of ANSSVm. Therefore, the first cleaved product of the ANSSVm polyprotein is likely to be an aberrant P3 protein fused with the truncated HC-Pro1 N-terminus (Figure 1).

### Construction of GFP-tagged infectious cDNA clones of ANSSVm

**FIGURE 2:**
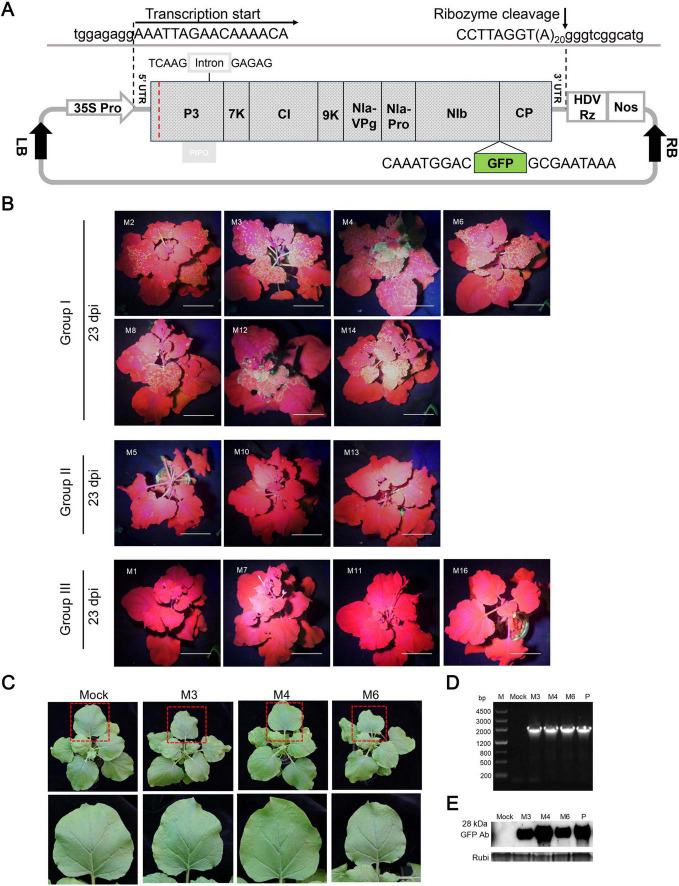
Systemic latent infection of ANSSVm in *N. benthamiana* via agroinoculation of a GFP-tagged infectious cDNA clone of ANSSVm. **(A)** Schematic representation of the structure of the GFP-tagged infectious cDNA clone of ANSSVm. The full-length cDNA of ANSSVm with a poly(A) tail of 20 nt was placed between an enhanced CaMV 35S promoter (35S Pro) and a self-cleaving HDV ribozyme (HDV Rz) followed by a NOS terminator (Nos) to generate viral transcripts that would have 5’ and 3’ termini identical with ANSSVm genomic RNA. An intron of the *PvNIR* gene was inserted into the P3 gene to stabilize the construct. The GFP coding sequence was integrated between NIb and CP following the proteolytic processing strategy of potyvirids to allow viral expression of GFP. Viral sequences were shown in uppercase while vector sequences were written in lowercase. The red dotted line indicates the junction between the residual HC-Pro1 and P3. LB, T-DNA left border; RB, T-DNA right border. **(B)** Infectivity test of 18 individual clones of pANSSVmG in *N. benthamiana* at 23 dpi. The representative plants were photographed under a hand-held blue light lamp. Individual clones were empirically divided into three groups based on differential GFP expression. Images of plants inoculated with systemic defective clones M9, M15, M17, and M18 (all belonged to Group III) were not shown due to the limitation of figure size. Scale bar = 4 cm. **(C)** Latent infection of ANSSVm in *N. benthamiana*. Photos were shown for representative plants inoculated with clones M3, M4, or M6 (all belonged to Group I that mediated strongest GFP expression) at 11 dpi. The closer view of an upper leaf (indicated by red box) was shown in the corresponding lower panel. Mock, the negative control inoculated with sterile inoculation buffer. **(D)** RT-PCR detection of ANSSVm in *N. benthamiana*. All inoculated plants were tested for the presence of ANSSVm in the upper systemic leaves by RT-PCR with the primer pair GFP-F/ANSSV-6R-3’UTR (Supplementary Table S1) at 1 mpi. Representative results were shown for plants inoculated with clones M3, M4, or M6. M, DNA marker, with sizes of DNA fragments indicated at the left; Mock, the negative control inoculated with sterile inoculation buffer; P, positive control: the plasmid of the M4 clone. **(E)** Western blot detection of GFP in ANSSVm-infected *N. benthamiana*. GFP protein in the upper leaves was detected with anti-GFP antibodies (GFP Ab) at 1 mpi. Coomassie Brilliant Blue (CBB) staining of the Rubisco large subunit (Rubi) served as the loading control. Mock, the negative control inoculated with sterile inoculation buffer; P, positive control: 16c transgenic *N. benthamiana* that constitutively expressed GFP protein.

Previous studies have identified *N. benthamiana* as a compatible host of ANSSV, with both HC-Pro1 and HC-Pro2 being essential for systemic infection (Qin et al., 2020). To assess the infectivity of ANSSVm, a GFP-tagged infectious cDNA clone of ANSSVm was constructed based on the GFP expression vector design of ANSSV-HNBT (Qin et al., 2020). The full-length ANSSVm cDNA, including a 20-nt poly(A) tail, was inserted between an enhanced CaMV 35S promoter and a self-cleaving HDV ribozyme (Been, 1994), followed by a NOS terminator. This design ensured RNA transcripts with the 5’ and 3’ UTRs identical to the ANSSVm genomic RNA. An intron was introduced into the P3 gene to stabilize the construct, and the GFP coding sequence was integrated between NIb and CP, following the proteolytic processing strategy of potyvirids, enabling viral expression of GFP. The resulting vector was designated as pANSSVmG (Figure 2A).

To account for potential aberrant nucleotides introduced during PCR amplifications and native nucleotide variants in the viral population that could affect clone infectivity (Peremyslov et al., 1998; Satyanarayana et al., 1999; Chen et al., 2021), 18 clones of pANSSVmG (denoted M1–M18) were randomly selected and preliminarily verified by restriction enzyme digestion. These clones were agroinfiltrated into *N. benthamiana* leaves, and the inoculated plants were monitored for GFP fluorescence and viral symptoms. ANSSVm presence in the upper leaves was further confirmed by RT-PCR and Western blotting using anti-GFP antibodies at 1 month post-inoculation (mpi). Among three independent trials, these 18 clones exhibited differential infectivity. Sparse fluorescent spots first appeared in the systemic leaves of plants inoculated with clone M4 at 8–9 days post-inoculation (dpi), making it the earliest to establish systemic infection in repeated trials. By 9–13 dpi, six clones (M2, M3, M6, M8, M12, and M14) demonstrated systemic infection with fluorescence signals progressively increasing in frequency and intensity, stabilizing around 25 dpi (Figure 2B), akin to ANSSV-HNBT (Qin et al., 2020). In contrast, clones M5, M10, and M13 exhibited scattered fluorescent spots in the upper leaves at 15–17 dpi, with minimal signal increase over time. The remaining eight clones (M1, M7, M9, M11, and M15–M18) showed no systemic infectivity and were deemed defective (Figure 2B). Based on these observations, the clones were categorized into three groups (Figure 2B): Group I: High infectivity, spreading to systemic leaves by 13 dpi and producing abundant fluorescent foci (M2, M3, M4, M6, M8, M12, and M14). Group II: Limited infectivity, reaching systemic leaves after 15 dpi with sparse fluorescent foci (M5, M10, and M13). Group III: Defective clones that failed to establish systemic infection (M1, M7, M9, M11, M15–M18).

Fluorescence in systemic leaves mediated by Group I and Group II clones remained stable after 25 dpi and persisted for at least one additional month (data not shown). Infection by ANSSV-HNBT typically induces vein clearing, foliar chlorosis, and leaf distortion in *N. benthamiana* (Qin et al., 2020). However, all plants inoculated with ANSSVm clones appeared symptomless and indistinguishable from mock-inoculated controls, suggesting that ANSSVm infection in *N. benthamiana* is latent (Figure 2C). Following the same experimental procedures, we constructed a GFP-tagged full-length infectious clone of another ANSSV isolate, ANSSV1 (GenBank PV657107), which induced severe necrotic spindle-spots in affected areca seedling. The 9,437 nt genome of ANSSV1 encodes a polyprotein of 3,019 aa. Its genomic organization is identical to that of ANSSV-HNBT, with identical 5’ UTR and 3’ UTR lengths and coding sequences for the proteolytically cleaved mature protein, sharing 98.78% nt identity (115 mismatches) between their genomes and 99.54% aa identity (14 mismatches) between their polyproteins. The ANSSV1 infectious clone was able to establish systemic infection in *N. benthamiana* via agroinoculation. Similar to the case of ANSSVm, ANSSV1 infection caused indistinguishable viral symptoms (Supplementary Figure S1).

### Amino acid substitutions in CP and P3 affecting viral intercellular movement

**FIGURE 3:**
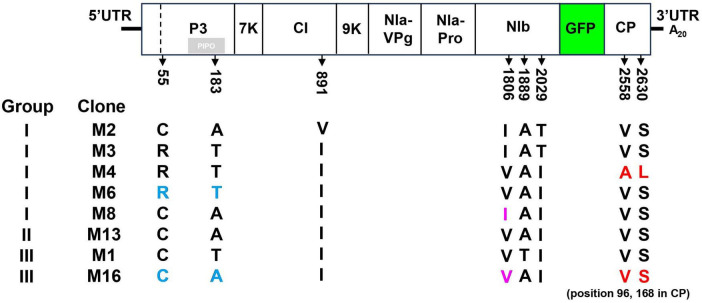
Multiple sequence alignment of amino acid sequences of representative pANSSVmG clones exhibiting differential infectivity. Eight representative clones of pANSSVmG: M2, M3, M4, M6, and M8 from Group I, M13 from Group II, and M1 and M16 from Group III were fully sequenced by next generation sequencing and comparatively analyzed. Multiple sequence alignment of the complete polyprotein ORF (including the exotic GFP) was employed to reveal a total of eight aa polymorphism: C/R^55^ and T/A^183^ in P3, I/V^891^ in CI, V/I^1806^, T/A^1889^, and I/T^2029^ in NIb, and V/A^2558^ and S/L^2630^ in CP. V/A^2558^ and S/L^2630^ of the polyprotein correspond to V/A^96^ and S/L^168^ of CP. Amino acid substitutions dissected in this work were highlighted.

To investigate the motifs or residues responsible for the differential bioactivity of pANSSVmG clones, eight representative clones—M2, M3, M4, M6, and M8 from Group I; M13 from Group II; and M1 and M16 from Group III—were fully sequenced and comparatively analyzed. Multiple sequence alignment of the complete ANSSVm-GFP polyprotein ORF revealed eight amino acid (aa) variations among the clones: C/R^55^ and T/A^183^ in P3, I/V^891^ in CI, V/I^1806^, T/A^1889^, and I/T^2029^ in NIb, and V/A^2558^ and S/L^2630^ in CP (Figure 3). Notably, no single aa substitution correlated with the compromised infectivity of M1 or M16. Interestingly, M13 (Group II) and M16 (Group III) encoded identical polyproteins (Figure 3), implying that nucleotide (nt) polymorphisms may influence their infectivity through synonymous mutations.

In repeated inoculation trials, GFP fluorescence consistently appeared first in M4-inoculated plants, at least 1 day earlier than that induced by other Group I clones. This observation suggested that M4 may possess enhanced abilities for cell-to-cell or systemic movement. Sequence alignment revealed that M4 has two unique residues—A^2558^ and L^2630^ in the polyprotein—corresponding to A^96^ and L^168^ in CP, whereas other clones retained the conserved residues V^96^ and S^168^ in CP (Figure 3). Given that the potyviral CP is critical for intercellular movement (Martínez-Turiño and García, 2020), these residues (V/A^96^ and S/L^168^) were hypothesized to be key determinants of intercellular movement in ANSSV CP.

**FIGURE 4:**
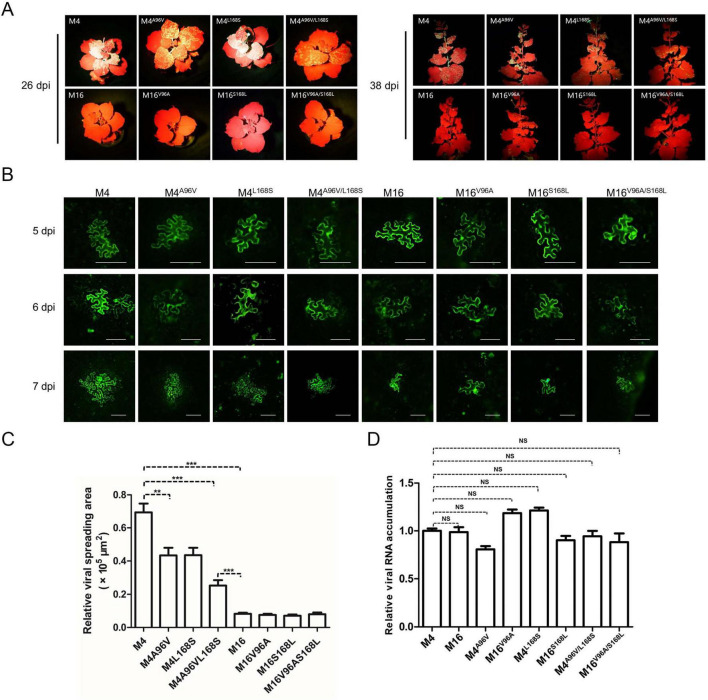
The rare A^96^ and L^168^ of M4-encoded CP affect viral intercellular movement. **(A)** Infectivity test of pANSSVmG clones M4, M16, and their point mutants in *N. benthamiana*. Agrobacterium strain GV3101 was used for agroinoculation. The representative images were taken under a hand-held blue light lamp. **(B)** Fluorescence microscopy analysis of the intercellular movement of pANSSVmG clones M4, M16, and their point mutants in *N. benthamiana* epidermal cells. Scale bar = 100 μm. **(C)** Statistical analysis of the size of viral spreading area at 5 dpi calculated by ImageJ. Data are mean ± *SEM* of three biological replicates. NS, not significant. **p < 0.01; ***p < 0.001. **(D)** Replication analysis of ANSSVm constructs in *N. benthamiana* epidermal cells. Total RNA was extracted from infiltrated leaf patches at 5 dpi and viral RNA was quantified by RT-qPCR with a primer set targeting CP (Supplementary Table S1). Data are mean ± *SEM* of three biological replicates. NS, not significant.

To test this hypothesis, point mutation constructs were analyzed. Using site-directed mutagenesis, two key sites—V/A^96^ and S/L^168^—were individually or simultaneously exchanged in M4 and M16 binary vectors, generating M4^*A*96*V*^, M4^*L*168*S*^, M4^*A*96*V*/*L*168*S*^, M16^*V*96*A*^, M16^*S*168*L*^, and M16^*V*96*A*/*S*168*L*^. The infectivity of these constructs was tested by Agroinfiltration of *N. benthamiana*. All M4 derivatives remained systemically infectious and expressed GFP, albeit with weaker fluorescence in M4^*A*96*V*/*L*168*S*^-inoculated plants (Figure 4A). Conversely, M16 derivatives failed to establish systemic infections, similar to wild-type M16 (Figure 4A). Fluorescence microscopy was employed to evaluate intercellular movement. Transformed agrobacterium cultures were diluted to an OD_600_ of 0.0001 before infiltration to obtain separated primary infected cells that allowed surveillance of viral cell-to-cell movement. At 5 dpi, GFP fluorescence was detected in primary infected cells for M4, M6, and their derivatives (Figure 4B). At 6 dpi, M4-infiltrated leaves showed both strongly fluorescent primary infected cells and neighboring cells with weaker fluorescence, indicating the onset of viral intercellular movement. However, all M4 mutants exhibited delayed movement, with M4^*A*96*V*/*L*168*S*^ showing the most pronounced delay (Figure 4B). M16 and its derivatives were confined to the primary infected cells and failed to move intercellularly, consistent with their inability to establish systemic infection (Figure 4B). To rule out replication-related effects, viral RNA levels were quantified via RT-qPCR at 5 dpi. No significant differences in viral RNA accumulation were observed among M4, M6, or their derivatives (Figure 4D). These findings suggest that the unique residues A^96^ and L^168^ in M4-encoded CP enhance ANSSVm cell-to-cell movement.

**FIGURE 5:**
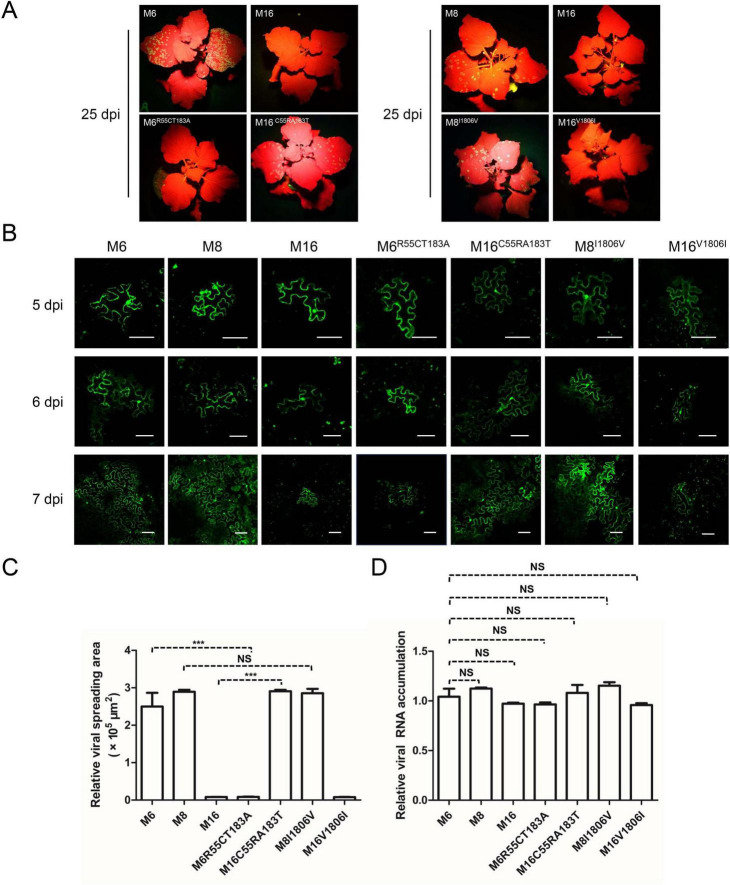
C/R^55^ and/or T/A^183^ in P3 is crucial for viral intercellular movement. **(A)** Infectivity test of pANSSVmG clones M6, M8, M16, and their point mutants in *N. benthamiana*. Agrobacterium strain LBA4404 was used for agroinoculation, and it mediated faster viral infection than that mediated by strain GV3101 (used in experiments for Figure 4). The representative images were taken under a hand-held blue light lamp. **(B)** Fluorescence microscopy analysis of the intercellular movement of pANSSVmG clones and their point mutants in *N. benthamiana* epidermal cells. Scale bar = 100 μm. **(C)** Statistical analysis of the size of viral spreading area at 5 dpi calculated by ImageJ. Data are mean ± *SEM* of three biological replicates. NS, not significant. ***p < 0.001. **(D)** Replication analysis of ANSSVm constructs in *N. benthamiana* epidermal cells. Total RNA was extracted from infiltrated leaf patches at 5 dpi and viral RNA was quantified by RT-qPCR with a primer set targeting CP (Supplementary Table S1). Data are mean ± *SEM* of three biological replicates. NS, not significant.

Aside from the CP residues, the comparison between M6 and M16 suggests that C/R^55^ and T/A^183^ in P3 could affect intercellular movement. Likewise, the comparison between M8 and M16 suggests that V/I^1806^ in NIb may be another key residue (Figure 3). To test the effects of these residues on viral intercellular movement, point mutation constructs of M6^*R*55*C*/*T*183*A*^, M16^*C*55*R*/*A*183*T*^, M8^*I*1806*V*^, and M16^*V*1806*I*^ were constructed and analyzed with the same procedure described above. The infectivity of these constructs was tested by Agroinfiltration of *N. benthamiana*. The substitution of V/I^1806^ between M8 and M16 did not affect intercellular movement (Figure 5). However, simultaneous substitution of C/R^55^ and T/A^183^ in P3 between M6 and M16 reversed their abilities to move between cells: M6^*R*55*C*/*T*183*A*^ was restrained in the primary infected cells, while M16^*C*55*R*/*A*183*T*^ acquired cell-to-cell movement (Figure 5). These findings suggest that C/R^55^ and/or T/A^183^ in P3 is crucial for the cell-to-cell movement of ANSSVm.

### The V/A^96^ and S/L^168^ substitutions in CP did not affect its interactions with host proteins

**FIGURE 6:**
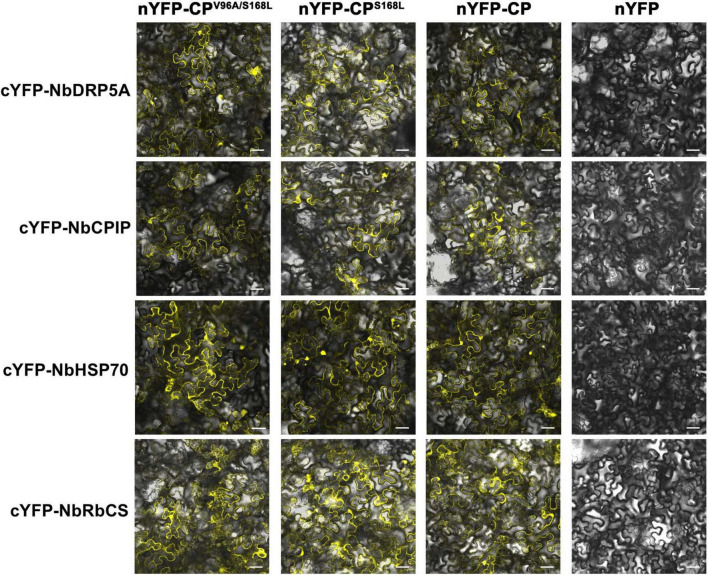
Screening of ANSSV CP-interacting proteins in *N. benthamiana*. The interactions between the consensus CP (V^96^/S^168^), CP^V96A/S168L^ (M4-encoded CP), or CPS^168L,^ and four potential CP-interacting host factors of *N. benthamiana* were preliminarily examined via BiFC assays. NbDRP5A, the *N. benthamiana* homolog of GmSDL5A (GenBank xxx, submitting); NbCPIP, the *N. benthamiana* homolog of GmCPIP (GenBank xxx, submitting); NbHSP70, a heat shock protein 70 of *N. benthamiana* (GenBank KX912913); NbRbCS, the Rubisco small subunit of *N. benthamiana* (GenBank MK070896). For all images, the corresponding N-terminal YFP (nYFP) vector and C-terminal YFP (cYFP) vector used were indicated at the top and at the left, respectively. The original empty vector nYFP served as the negative control. YFP fluorescence was documented by confocal microscopy at 3 dpi. Scale bar = 50 μm.

The potyviral coat protein (CP) is a multifunctional protein involved in nearly all stages of the viral life cycle (Martínez-Turiño and García, 2020). Several host factors have been reported to interact with potyviral CP, facilitating various phases of infection (Hofius et al., 2007; Lõhmus et al., 2017; Wu et al., 2018; Zong et al., 2020; Qin et al., 2024). To investigate whether ANSSV CP interacts with these proteins or their homologs in *N. benthamiana*, and how the V/A^96^ and S/L^168^ substitutions in CP affect such interactions, four known CP-interacting proteins were selected: the endocytosis dynamin-like protein 5A of soybean (GmSDL5A), a DnaJ protein of soybean (GmCPIP), a heat shock protein 70 (NbHSP70), and the Rubisco small subunit (NbRbCS) of *N. benthamiana*. Homologs of GmSDL5A and GmCPIP in *N. benthamiana*—designated as NbDRP5A and NbCPIP, respectively—were identified through BLAST searches against the Sol Genomics Network (SGN) database (Fernandez-Pozo et al., 2015) and cloned using corresponding primers (Supplementary Table S1). The interactions between the consensus CP^96V/168S^, CP^*V*96*A*/*S*168*L*^ (M4-encoded CP) or CPS^168L^ and these host factors were preliminarily evaluated via bimolecular fluorescence complementation (BiFC) assays. BiFC results showed that all three forms of CP interacted with NbDRP5A, NbCPIP, NbHSP70, and NbRbCS (Figure 6), indicating that the V/A^96^ and S/L^168^ substitutions in CP did not affect its interactions with these host proteins.

## Discussion

ANSSV encodes HC-Pro1 and HC-Pro2, which are essential for establishing systemic infections (Qin et al., 2020). Notably, HC-Pro2 interacts with viral proteins CI and CP, as well as the host Rubisco small subunit (RbCS), forming a plasmodesmata-localized protein complex that facilitates viral intercellular movement (Qin et al., 2024). These findings underscore the critical roles of HC-Pro1 and HC-Pro2 in viral infection. However, our study identified a mild ANSSV isolate, ANSSVm, with a truncated genome of 7,868 nt, lacking both HC-Pro1 and HC-Pro2, yet capable of establishing systemic infection in *N. benthamiana*. This unexpected result highlights the complexity of potyvirids-host interactions and opens avenues for investigating alternative mechanisms driving viral movement and systemic infection of potyvirids.

To explore these mechanisms, 18 GFP-labeled ANSSVm infectious clones were agroinfiltrated into *N. benthamiana*, exhibiting varying infectivity levels (Figure 2). Sequencing and alignment of the complete ANSSVm-GFP polyprotein ORF revealed amino acid (aa) variations among the clones (Figure 3). Strikingly, some clones encoding identical polyproteins displayed distinct infectivity, suggesting that nucleotide polymorphisms may influence their infectivity. Among these variations, residues V/A^96^ and S/L^168^ in CP, as well as C/R^55^ and/or T/A^183^ in P3 emerged as key determinants of viral intercellular movement (Figures 4, 5). Simultaneous substitution of C/R^55^ and T/A^183^ in P3 between M6 and M16 reversed their abilities to move between cells, suggesting that R^55^ and T^183^ is functional while C^55^ and A^183^ being movement-defective. However, two systemic infectious clones M2 and M8 also possess C^55^ and A^183^. These two residues may be crucial for P3 to interact with other viral proteins, while working in certain combinations (like C^55^/A^183^—I^1806^, or R^55^/T^183^—V^1806^, Figure 3). Moreover, nucleotide polymorphisms may influence viral infectivity through synonymous mutations as revealed by the comparison between M13 and M16 (Figure 3). Given the complexity of combinations of nucleotides and aa residues, these findings warrant further investigation.

In the construction of infectious clones of viruses with large genomes, it is difficult to synthesize the full-length viral genome in a single step. Instead, the large genomes would be cloned as fragments of 2–4 kb and serially assembled by multiple ligation and transformation steps, or by one-step assembly with all the fragments. In this process, possible defectives within the native viral population, errors during RT-PCR, and propagation of plasmids in *E. coli* could contribute to compromised infectivity of the resulting constructs (Peremyslov et al., 1998; Satyanarayana et al., 1999; Chen et al., 2021). Plasmid stability could be somehow improved by using special *E. coli* strains, or by reducing the temperature for *E. coli* incubation (Orílio et al., 2014). In this work, the *E. coli* strain Stbl2 helped to stabilize the viral constructs and improved plasmid yield. During multiple experiments, we have sequenced the plasmids by Sanger sequencing or NGS sequencing. The results are consistently stable and no sign of modification by bacteria has been found. However, the risk that some SNPs might be introduced during propagation in *E. coli* could not be technically excluded.

The interactions between CP and host proteins are critical for intercellular movement and systemic infection of potyvirids. For instance, the dynamin-like protein GmSDL5A in soybean interacts with TuMV CP, influencing viral replication and movement (Wu et al., 2018). Similarly, the DnaJ protein GmCPIP enhances Soybean mosaic virus (SMV) infection via interacting with CP (Zong et al., 2020). Tobacco DnaJ-like proteins (NtCPIPs) interact with Potato virus Y (PVY) CP, functioning as susceptibility factors (Hofius et al., 2007). Additionally, heat shock proteins CPIP and HSP70 interact with Potato virus A (PVA) CP, coordinating with other host factors to regulate CP accumulation (Lõhmus et al., 2017). In ANRSV, the Rubisco small subunit (NbRbCS) interacts with CP and CI, acting as a scaffold for a complex facilitating intercellular movement (Qin et al., 2024). BiFC assays in this study demonstrated that the consensus CP (V^96^/S^168^), CP^*V*96*A*/*S*168*L*^ (M4-encoded CP), and CP^*S*168*L*^ interacted with NbDRP5A, NbCPIP, NbHSP70, and NbRbCS, suggesting that these amino acid substitutions in CP did not disrupt its interactions with host proteins (Figure 6). However, the potential impact of these mutations on virion assembly merits further investigation, particularly given the established requirement of intact virion formation for efficient viral movement in potyviruses (Dai et al., 2020). Our study identified several additional aspects requiring exploration: potential alterations in interactions with other movement-associated viral components such as CI proteins, possible effects on CP-CP self-association dynamics, and the observed correlation between ANSSVm clone nucleotide polymorphisms and infection efficiency. Further studies are needed to identify additional host factors and interactions crucial for systemic spread, potentially enabling targeted strategies to combat viral infections in plants.

Finally, the ANSSVm isolate, identified from a leaflet with mild symptoms, lacks the tandem HC-Pros and does not induce visible symptoms in *N. benthamiana* after agroinfiltration. ANSSV-HNBT and ANSSV1, encoding two HC-Pros, were both identified from areca palms with severe symptoms. HC-Pro2 encoded by ANSSV has been experimentally proved to be a viral suppressor of RNA silencing (VSR), combating the host antiviral defense (Qin et al., 2020). Heterologous expression of VSRs by viral vectors has been reported to enhance the symptom severity of the recombinant viruses (Díaz-Pendón and Ding, 2008), and these VSRs are therefore regarded as pathogenicity determinants. HC-Pro2 could be a pathogenicity determinant encoded by ANSSV. With this assumption, ANSSVm is a promising candidate for being engineered as a virus-induced gene silencing (VIGS) vector for areca palm. Although the genetic transformation of areca palm has not yet been achieved, this approach holds significant potential for gene characterization and breeding disease-resistant areca palm varieties.

## Data Availability

The datasets presented in this study can be found in online repositories. The names of the repository/repositories and accession number(s) can be found below: https://www.ncbi.nlm.nih.gov/genbank/, PQ776890 and PV657107.
