# Supplementary material for: Identification of a novel mild isolate of areca palm necrotic spindle-spot virus (ANSSVm) lacking two cysteine proteases (HC-Pro1 and HC-Pro2)
Source: Front Microbiol. 2025 Jul 15;16:1553892. doi: 10.3389/fmicb.2025.1553892 (PMC12303960; doi:10.3389/fmicb.2025.1553892)
Supplement: Supplementary file 2 [file Data_Sheet_1.docx]

**
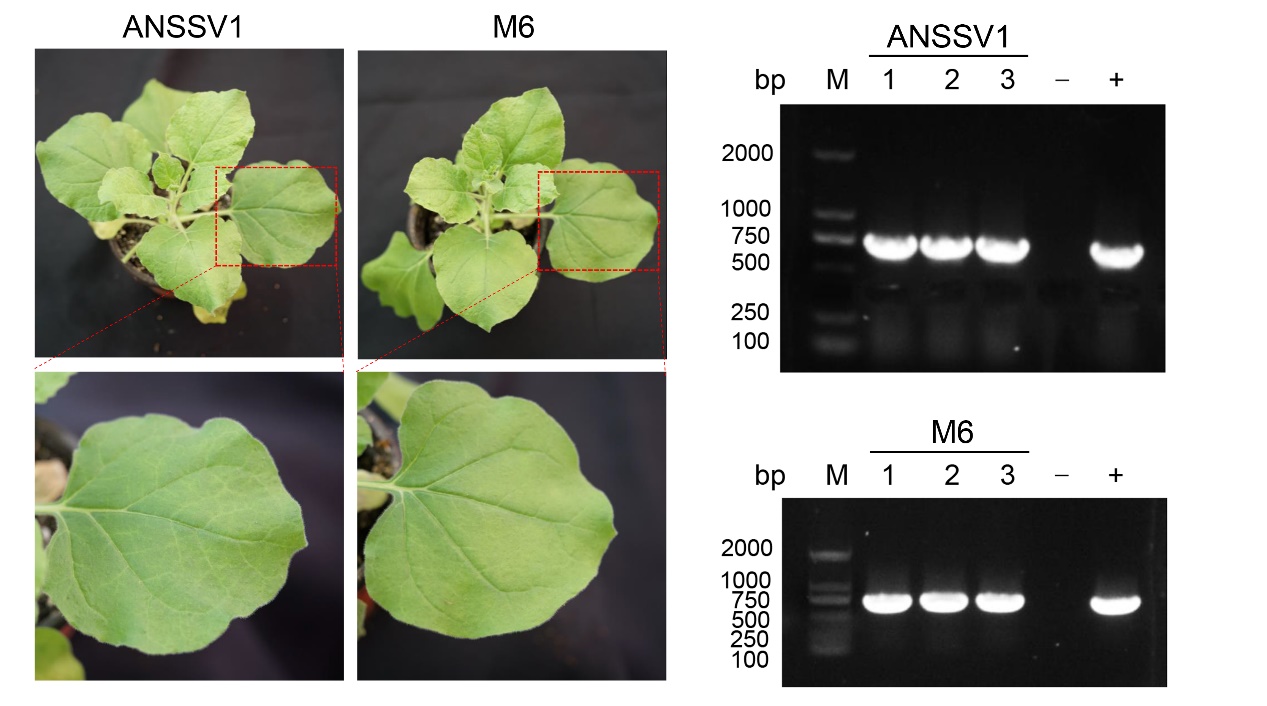
**

**Supplementary Figure 1.** Infectivity test of ANSSV1 (an isolate with full-length genome encoding two HC-Pros, causing severe symptoms in areca seedling) and ANSSVm-M6. Representative images were taken at 15 days post inoculation. The presence of ANSSV1 and ANSSVm in upper systemic leaves was confirmed by RT-PCR.
